# General Intelligence

**Published:** 1844-12

**Authors:** 


### GENERAL INTELLIGENCE.

*Trial for Malpractice.* Benjamin Bartlett *vs.* Solomon Blood.—This was an action for damages, brought by the plaintiff, in the Racine County District Court, October 30, 1844. It appeared by the testimony that Bartlett, about a year previously, while working on a house, fell, and the roof, covered with snow, fell upon him. He was carried to his own house and Dr. Blood sent for, who, on arriving, pronounced it a fracture "of the point of the shoulder," and for dressing, applied a pad between the arm and breast, a roller about both; and, according to some witnesses, one which passed from the elbow of the affected side to the opposite shoulder. This required replacing twice; and after the second replacement, about three weeks after the accident, the Doctor's attendance was discontinued, he alleges to have been dismissed. On examination of the arm, Oct. 29, 1844, we found the following appearances. The shoulder presented considerable fullness, the muscles not being very much atrophied for want of use. The external extremity of the clavicle was a little above the acromion, the superior surface of which was irregular, and had the appearance of having been slightly depressed at its point, but was immoveable. The arm could be raised to a horizontal position. The rotation was imperfect, and when raised to that extent the tendons of the pectoralis major, and of the latissimus dorsi and teres major muscles, were felt to be tense. The forearm could be extended so as to form an angle of 45°, with the line of axis of the humerus, and flexed a little beyond a right angle. From this latter circumstance, it had been supposed that the radius was dislocated forwards, but this part of the declaration was withdrawn before the trial. The hand was prone and could only be partially supinated, the tendons about the elbow were rigid and tense when efforts were made to perform extended movements. These were the only abnormal appearances. It was the opinion of most of the medical witnesses, that there had been a fracture of the acromion, which was well re-united, an opinion which we ourselves entertained. The loss of movements we attributed to the rigidity of the fibrous tissues, but whether the original injury was such as to have permitted of a perfect restoration of the member, *within a year*, we would not undertake to decide, nor do we think any one can do so. The perfect union of the acromion showed there were not great defects in the treatment of the fracture of it. The jury, however, seemed to think the Doctor was bound to restore the arm to its movements, and gave a verdict for the plaintiff of $300 and costs.

If we consider that the contusions must have been extensive, and that there may have been injury of the axillary plexus of nerves; that when a surgeon is dismissed he has often no means of proving it; that the movements of the member might be still improved by suitable treatment, we doubt not that Dr. Blood will have the sympathies of many members of the profession; for, if a surgeon is to pay all the damages which patients may sustain from such injuries, without their being called upon to show that they took suitable means for the restoration of the movements of the member, *when the surgeon was not in attendance*, we see no security for the rights of the latter. If instead of commencing a suit Mr. Bartlett had applied to a surgeon, we think ourselves justified in expressing the opinion, that there was still time for the restoration of many of the movements and uses of the member, and that, even now, when a year has elapsed, much might be done by judicious and continued treatment, to restore it.

D. B.

---

*Death of Dr. Samuel Forry.*—Although the death of this gentleman was noticed in our necrological record last week, we are prompted, by feelings of respect for his memory, to recur again to the melancholy event. Those who were conversant with Dr. Forry's condition in his last sickness, unhesitatingly say that he was a martyr to severe study. The brain was over-worked by laborious investigations, and he fell, in the beginning of his usefulness. His organization appeared to be feeble, and liable to be easily deranged by prolonged mental activity. It is probable that the exposed life he led in the Florida war, while holding a medical commission in the Army, contributed to weaken the physical energies of a naturally frail body, and lessened the resisting powers of the system to the attacks of acute disease. In person, Dr. Forry was of middling stature; his figure was slight, with an expression in his countenance that was indicative of the man of thought. In the circles of medical sciences vital statistics seemed to be the province which afforded him the most satisfaction. Perhaps no one was ever more thoroughly devoted to investigations on the effects of climate, than himself. The work produced by him on this subject during the last season, is the one which will most favorably transmit his name to posterity. In it the peculiar bias of his active mind was exhibited. For analyzing great principles, and explaining laws and phenomena connected with this department of science, he had no superior in this country. As a journalist, and chronicler of the claims and fame of others, he had scarcely time, since the commencement of an editorial career, to exhibit all the talent we considered him capable of exercising. There was a fairness and spirit of candor in his leading editorial comments, that never provoked others to war against the editor, however much some might have objected to the freedom of correspondents.

A meeting of some of the physicians of New York was recently held, at which resolutions were passed in relation to the early death of Dr. Forry, exceedingly creditable to them. May the monument they propose to raise over his grave speedily mark the spot; and may those who knew him, emulate his virtues, that they may, like him, live respected and die lamented.—*Boston Med. Journal.*

---

A letter from Dr. JNO. EVANS arrived after the article referred to therein had gone to press. His request could not, therefore, by any possibility, be complied with. ED.

